# The Challenges of Gender Diversity in Boards of Directors: An Australian Study with Global Implications

**DOI:** 10.1002/gch2.202400259

**Published:** 2024-12-25

**Authors:** Suzanne Young, Karen Farquharson, Daswin De Silva, Paul Mather

**Affiliations:** ^1^ La Trobe Business School La Trobe University Melbourne 3086 Australia; ^2^ School of Social and Political Sciences The University of Melbourne Parkville 3010 Australia; ^3^ Centre for Data Analytics and Cognition La Trobe University Melbourne 3086 Australia

**Keywords:** australia, boards, diversity, gender, networks, quotas, upper echelon theory

## Abstract

Despite gender diversity being driven by institutional bodies, companies have been relatively slow to diversify. In this study, it is investigated that how Australian boards select new directors, and how those selection processes affect their recruitment of women. In‐depth interviews are conducted of those with first‐hand experience of board appointments, followed by the thematic analysis and the application of natural language processing techniques to identify emotions and sentiment associated with these themes. The findings indicate that boards are adopting a social rather than rational approach to board selection. They are using networks, recruitment agencies, skills matrices and pools which on the surface appear to broaden the diversity of board members. But if they are not actively seeking gender diversity these methods can still limit diversity. For women, the lack of progress and barriers of access are resulting in high intensity of negative emotions. A key contribution of the research is the intersection of social approaches to board appointment and social identity theory with the dynamics of gender. Boards need to prioritize diversity for it to be achieved. There is a need for more active methods of recruitment and expansion of the networks and pools where directors are traditionally sought. Institutions can drive change through increasing targets and requiring enhanced reporting.

## Graphical abstract

The process of selecting new members to boards of directors is social rather than rational. Although, methods such as recruitment agencies and skills matrices are being used with the intention of broader reach, most boards are still not actively seeking diversity. Female participants expressed high intensity of negative emotions and a preference for quotas to address the lack of progress.

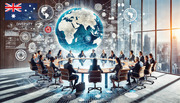

## 1 Introduction

Normatively, there is a widely held view that gender diversity on boards should be achieved by firms for reasons of fairness, ethics and participation.^[^ ^1^, ^2^, ^3^ ^]^ Moreover, there is much evidence as to the business benefit in terms of improving board, and ultimately organizational, performance.^[^ ^4^ ^]^ Research papers, governance reports, best practice guidelines, all point to diversification as a key objective in governance reforms. Some countries such as Norway Belgium, Denmark, and France, amongst others, legislate to improve board diversity, whilst others, such as Canada and Australia, opt for voluntary guidelines.^[^ ^5^, ^6^, ^7^ ^]^ Increasingly we also see key institutional investors, rating agencies and regulators pressuring companies to adopt diversity targets and measures.^[^ ^3^, ^8^ ^]^

Even so there are some mixed findings around company financial performance (see ref. [9] for a review). Empirical research has found that the link between gender diversity and firm performance follows a U‐shaped curve and it is only after achieving a critical mass of 30% women that it is associated with higher firm performance than purely male boards.^[^ ^10^ ^]^ Some research has also found evidence of increased conflict leading to slower decision‐making associated with appointing women on boards.^[^ ^11^ ^]^ Interestingly, a study of global gender diversity found that there is a positive relationship between gender diversity and firm performance when voluntary approaches to increasing gender diversity are used, compared to a negative association when regulation is used.^[^ ^12^ ^]^ Although others have found that mandatory regulation is key.^[^ ^13^ ^]^

Notwithstanding, the preponderance of research on this issue reports positive outcomes associated with board gender diversity. Whilst not exhaustive, some examples include findings that gender diverse boards improve independence, competitive advantage and earnings,^[^ ^14^, ^15^, ^16^ ^]^ improve decision making, through innovation and creativity,^[^ ^17^ ^]^ provide new insights,^[^ ^1^ ^]^ and minimize group decision‐making biases.^[^ ^18^ ^]^ Moreover, having women on boards has been found to improve ethical decision‐making,^[^ ^19^ ^]^ and affords for discussion of tougher issues and more informed decisions.^[^ ^20^ ^]^

The numbers of women on boards differs significantly between and within countries^[^ ^3^, ^8^, ^21^ ^]^ and generally remains low.^[^ ^7^, ^22^ ^]^ Given the evidence as to gender diverse boards improving fairness and equity, we are interested in why companies remain slow to diversify their boards. Even though many countries such as Norway, France, Sweden, Finland, New Zealand and Belgium in 2018 had over 30% of board seats held by women, other countries such as Greece, Indonesia, Taiwan, Brazil, and many others had less than 10%.^[^ ^5^ ^]^ In Australia women on ASX200 (ASX200 is a market‐capitalisation weighted and float‐adjusted stock market index of the largest 200 stocks listed on the Australian Securities Exchange) boards only exceeded 5% in the past seven years and only passed 10% in 2020.^[^ ^18^ ^]^ Wright, Cortese, Al_Mamun and Ali (2023) found that women board members in Australia in 2023 were more likely to be interlockers with higher average betweeness scores compared to male board members. De Cabo et al.^[^ ^23^ ^]^ found that heavily male‐dominated boards resist the appointment of women board members which slows down the overall progress in increasing gender diversity. Other possible explanations for the slow progress include the voluntary nature of regulation around board diversity,^[^ ^7^ ^]^ unfavorable perceptions of women leadership compared to men,^[^ ^3^, ^8^, ^24^ ^]^ gender discrimination^[^ ^22^ ^]^ and group decision‐making biases.^[^ ^23^ ^]^

This research aims to understand how Australian boards select new directors, and how those selection processes affect their recruitment of women, through carrying out in‐depth interviews with those with first‐hand experience. The voluntary nature of the Australian governance environment provides an important context for this research. Much of the extant research is situated in the US, where progress has been slow^[^ ^8^ ^]^ resulting in many firms having a single woman director.^[^ ^9^, ^25^ ^]^ We explore the social processes of board selection and social identity theory^[^ ^26^ ^]^ to investigate why there is still diversity outcomes that would not be expected with the institutional noise surrounding the importance of diversity on boards. There has been a range of voluntary institutional drivers with the Australian Institute of Company Directors having instituted a voluntary target of women on boards of 30% by 2018, and the ASX Corporate Governance Council recommending that listed companies report on the gender proportions of board positions, in senior executive roles and across the organization yearly.^[^ ^5^, ^18^ ^]^ Advocacy groups such as Women on Boards, Male Champions of Change and Chief Executive Women have been calling for increases in women on boards over many years.^[^ ^27^ ^]^ This paper seeks to shed light into the processes companies in Australia utilize to increase board diversity. Through in‐depth interviews, we explore the drivers and barriers that impact decision‐making surrounding increasing diversity on boards. It brings these to the fore by exploring the process of board member selection and appointment, arguing that the decision to seek diversity and the recruitment approach taken are the two key points where diversification is facilitated or blocked. Boards must genuinely want to diversify, and recruitment practices must be adopted that enable the identification and selection of qualified candidates.

This paper is motivated by the need to extend the extant literature by examining many dimensions around the decision‐making through the qualitative approach enabling us to draw a far greater range of insights than those reported by studies using secondary data. Further, the use of recent advances in data analytics (including sentiment analysis) on transcripts facilitates the identification of the type and intensity of a continuum of emotions. This adds a significant dimension to the thematic textual analysis, allowing the research to provide insights into the emotional responses of the interviewees. Given the preponderance of empirical governance work on gender our study aims to explore the gap in understanding the opinions and decision‐processes of key governance actors as well as those who are making the board selection decisions. De Casbo et al.^[^ ^23^ ^]^ argued that gender diversity on boards disseminates through social networks and that men may seek those in their in‐group to maintain their own status and prestige. Indeed, our research builds on this and demonstrates through the statements of those very governance actors that men as key decision‐makers are using established networks and recruitment methods that reinforce the appointment of those in their own likeness and, even though arguing that they use “rational” rather than “social” approaches to recruitment, are indeed using established institutional structures and networks to maintain the status quo.

## 2 Related Work

Despite diversity having been the subject of much commentary and research for decades,^[^ ^2^, ^3^, ^9^, ^28^ ^]^ there is minimal research on board diversification that investigates the opinions of board members and Chief Executive Officers (CEOs) themselves,^[^ ^18^ ^]^ through what is referred to as elite interviewing.^[^ ^29^ ^]^ Indeed De Cabo et al.^[^ ^23^ ^]^ recently called for interviews with company directors to capture the process of board appointment decisions. This gap is not surprising given the difficulties in accessing company directors,^[^ ^22^ ^]^ particularly with concerns from the participants about how confidentiality will be managed^[^ ^2^ ^]^ in relatively small director communities such as Australia. Since these are the people who make the decisions as to board membership the exploration of their views is an important contribution.^[^ ^18^ ^]^

### 2.1 Gender Quotas and Regulation

At the national level in many countries the state is driving board diversity through the institution of gender quotas and regulation.^[^ ^3^, ^6^, ^30^ ^]^ In many countries it is accepted generally that women need to comprise a minimum percentage of board members, typically between 30 and 40%.^[^ ^9^, ^31^ ^]^ Some countries, such as the UK and Australia, have recommended board diversity through their voluntary governance principles. In the absence of quotas, external pressures can compel firms to introduce the first female director.^[^ ^22^, ^25^ ^]^ However, that is often as far as a board goes in diversifying: indeed the likelihood of appointing an additional female director falls when one female director is on the board.^[^ ^32^ ^]^ In such cases it is argued that it is the organizational dynamics that are more salient to adding more women to the board.^[^ ^33^ ^]^ At the organizational and board levels, it has been found that larger boards tend to exhibit more diversity;^[^ ^34^, ^35^ ^]^ and larger companies in Australia do seem to be improving diversity.^[^ ^1^, ^36^ ^]^ The number of ASX300 companies with at least 30% women directors tripled between 2016 and 2020, whilst the number of companies with zero or one female directors halved over the same time period.

### 2.2 Board Selection Process

Board members are selected using networks and external recruiters, and appraised using skills matrices.^[^ ^25^ ^]^ Even so board selection literature emphasizes the social processes and biases that may influence board selection.^[^ ^37^, ^38^ ^]^ The person nominated to the board is often evaluated against the characteristics of the exiting board member based on the desire to affiliate with those like themselves or other social elites.^[^ ^38^ ^]^ It is presumed that the directors most likely to be appointed are those who reflect boardroom norms.^[^ ^39^ ^]^ CEO power is used to influence board selection as they may desire directors who are disinclined to closely monitor management.^[^ ^40^ ^]^ Westphal and Zajac^[^ ^41^ ^]^ found that CEOs with greater power in relation to the board, influence board selection by selecting directors with similar demographic characteristics to reinforce their power. Guildiken, Mallon, Fainshmidt, et al.^[^ ^25^ ^]^ note that Top Management Teams (TMTs) influence board gender diversity, and when TMTs are dominated by men, women directors are less likely to be selected (also see refs. [41, 42]). The decision makers also favor current and ex‐CEOs which exclude a lot of high‐quality capable women.^[^ ^25^ ^]^ The director nomination process provides an opportunity for directors to associate with, and ingratiate themselves with, those who will provide benefit to themselves.^[^ ^38^ ^]^ At the more micro level, formal educational attainment as well as business experience have been studied as antecedents of board selection.^[^ ^28^, ^35^ ^]^ Personal characteristics are also important and include skills and experiences, expertise and network ties.^[^ ^38^, ^43^ ^]^ Reputation and prestige of directors are forms of social capital that can serve as signals to investors of the firm's prestige and render it likely that such directors be appointed.^[^ ^44^ ^]^ Female directors network ties and board experience and interlinkages with other firms lead to future board roles^[^ ^21^ ^]^ whilst their lack of networks limits their board membership opportunities.^[^ ^45^ ^]^

The range of literature reviewed demonstrates that drivers arise from legislation and regulation, and national culture, values and norms, from industry and organizational characteristics, and from board structure and individuals who comprise the TMT and the board. The literature demonstrates that though the drivers are influential, it is the decision‐makers at the organization and board level that ultimately make the decision as to the search and recruitment methods used to develop a pool and then decide who is appointed to the board. Hence our paper focuses on interviewing key individuals who make and influence the decisions, and explores how the selection and recruitment is carried out.

## 3 Theoretical Framework

Drawing on the social perspective of board selection^[^ ^38^ ^]^ and social identity theory^[^ ^26^ ^]^ we argue that the selection of board members is a social rather than rational process, whereby board members are selected based on their congruence with boardroom norms, their reflection of the characteristics of the exiting or current board members, and their embeddedness in networks or other boards that by association deliver reputation and status to the board. Directors and CEOs are those who claim the expertise and knowledge to be able to make informed decisions and are likely to select and influence the selection of other board members that reflect their own backgrounds, characteristics and networks, through the lens of their own experiences, values, and personalities. As such they are subject to bounded rationality in their decision‐making, reflecting their own set of values, where decisions are made based on uncertain and complex information.^[^ ^26^ ^]^

Boards can limit managerial discretion, and hence CEOs gain more power if they can limit board activities and the boards exertion of power over organizational strategy. As such CEOs may desire to appoint directors who they can control, and who are similar in characteristics and values, and who are part of their own networks, to reduce uncertainty and maintain their own power. Finkelstein^[^ ^46^ ^]^ adds that leadership teams influence strategic behavior moreso when they hold more power, and hence they can influence board selection to those in their own likeness. For directors, their own networks are an important source of power, maintaining contact with the business elite and powerful coalitions.^[^ ^46^ ^]^ Social networks and connections which are dependent on interpersonal political factors and individual biases limit the appointment of new board members.^[^ ^47^ ^]^

**Figure 1:**
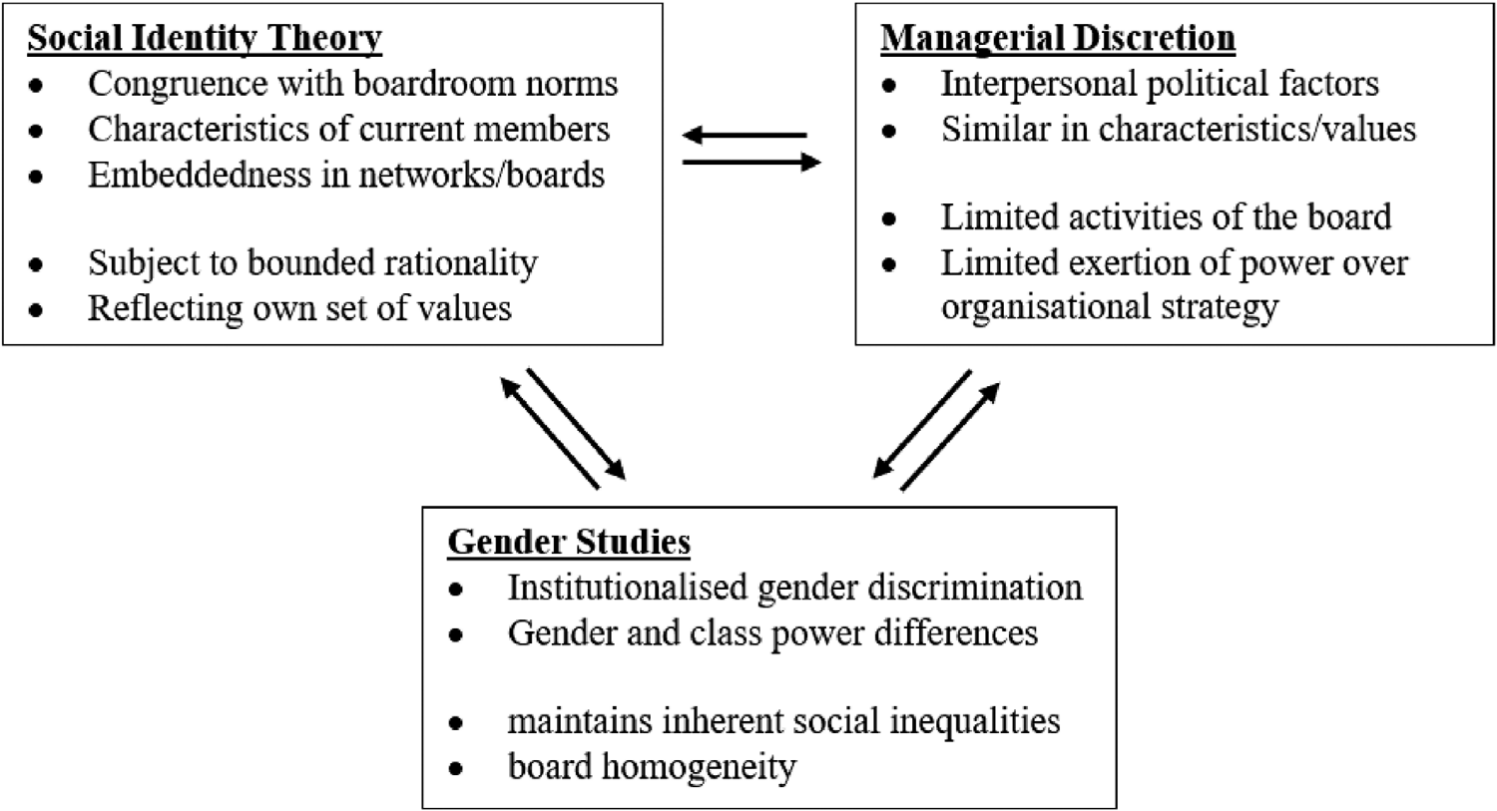
Diagrammatic representation of the theoretical framework.

Drawing on gender studies (such as ref. [48]), we argue that boards are dominated by men with members appointed from their own networks to maintain their power and thus maintain the inherent inequalities found in society and projected in organizations. Gender and class are important determinants of power differences in organizations^[^ ^48^ ^]^ and the source of organizational power are boards. Gender and class are interrelated as managers have traditionally been men, with lower levels of white‐collar workers being women.^[^ ^48^ ^]^ Even though this has changed with many managers now being women, at the ultimate decision‐making levels of organizations, namely at the board level, historical manifestations of gender and class remain. Indeed, Doldor and Vinnicome^[^ ^49^ ^]^ and Goyal, Kakabadse, Kakabadse and Talbot^[^ ^22^ ^]^ argue that selection decisions about suitability, relevant experience and merit and competency are gendered and hence board selection decisions are biased. As such institutionalized gender discrimination creates board homogeneity.^[^ ^22^ ^]^ Indeed gender discrimination has been found to limit women's board representation in Europe and the UK^[^ ^50^, ^51^ ^]^ and US^[^ ^23^ ^]^ and India^[^ ^52^ ^]^ and even where they do obtain representation implicit bias may continue.^[^ ^22^ ^]^ In board literature this body of work has implications for the way board selection decisions are made and how the directors and board members recruit potential members into their pool. **Figure** **1** presents a diagrammatic representation of this theoretical framework utilized in this study.

In the context of the research problem, this theoretical framework reveals that board member selection is a socially driven process, influenced by interpersonal networks, shared identities, and values of existing board members, which can perpetuate gender and class inequalities and limit diversity, thereby reinforcing organizational power structures. This social selection process, shaped by implicit biases and historical patterns of gendered and class‐based discrimination, leads to boards that are homogeneous and resistant to change, further entrenching power imbalances within organizations.

## 4 Research Methods

Despite a range of multi‐level drivers, from international and national institutions, organizations and boards, and strong business and normative reasoning for improving board diversity, progress toward gender equity on boards is slow. To better understand why this is the case in Australia, this study explores how Australian boards select new directors and how this in effect brings about gender diversity on boards. Our study focuses on board diversity in Australian companies and seeks to gain an understanding of this by examining the views of predominantly directors from a range of company boards by including large for‐profit companies, not‐for‐profit organizations and start‐up companies. Notwithstanding the differences in ownership and board structures across these organizational forms this exploratory study seeks to understand the opinions of a range of directors and key governance actors who influence the opinions and actions of directors. The issue of gender diversity on boards is not limited to one organizational form.

Given the nature of the research questions the research adopts an exploratory method using qualitative interviews to answer the research questions. We were interested in interviewing broadly to triangulate the data to reduce the effects of social desirability bias. We interviewed both men and women to explore the issue.^[^ ^2^ ^]^ A purposive sampling approach^[^ ^53^ ^]^ was used to access participants from a range of organizational forms. The research gained ethics approval from the University Ethics Committees and all respondents were treated confidentially.

Potential participants were identified using researchers’ and participants’ networks adopting snowball techniques (as per ref. [22]). A total of 41 interviews were undertaken between January August 2019 of which 21 were women. Interviews typically took 1 h but ranged from 35 to 90 min. The interviews were predominantly conducted by one of the authors to ensure consistency after initial piloting of the questions with interviewees by two of the authors. All interviews were digitally recorded. The interview transcripts were returned to the interviewees for checking before coding. We adopted a semi structured interview protocol providing each interviewee with the opportunity to tell the story of board diversity before moving to a more structured group of questions. Broad questions were posed around board diversity, what it means and how it is brought about.

**Table** **1** presents the breakdown between those respondents who were current directors or held recent directorships on Australian boards and those who were employed by other organizations. Additional information is provided in Appendix A.

**Table 1:** Participant demographics by classification.

Classification (n = 41)	Number	Gender (n = 41)	
		Male	Female
Directors	30^a)^	16^a)^	14
Institutional representatives	12^a)^	5^a)^	7
Total	42	21	21

NB: 41 interviews were conducted.

^a)^ One interviewee was both an institutional representative and director of a start up company.

**Figure 2:**
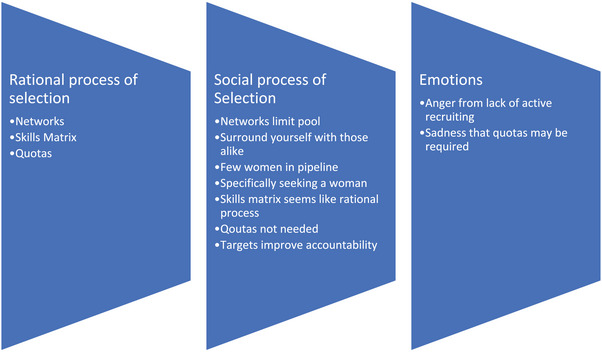
Research analysis.

We used an iterative process and thematic analysis comparing and analyzing the data.^[^ ^53^ ^]^ To develop a coding framework, interview transcripts were independently coded in NVivo and by three researchers who then met to refine the codes. The coding framework is shown in **Table** **2**. First order codes were networks, skills matrices, and quotas. Themes that emerged from the analysis are shown in Table 2. Even though the first order codes may on the surface demonstrate ways of bringing about gender diversity such as through networks, it was by examining the quotes in detail that we found that they may actually be referring to actions that limit diversity, such as where networks limit the pool from which board members are sought. **Figure** **2** illustrates the data analysis workflow.

**Table 2:** Coding framework.

Codes	Themes
Networks	*Networks limit pool* *Use of networks to surround yourself with those similar (limit diversity)* *Few women in pipeline* *Specifically seeking a woman* *Lack of active recruiting to find women brings about anger*
Skills Matrix	*Use of skills matrix seems like a rational process*
Quotas	*Quotas not needed‐ women already appointed on merit* *Targets improve accountability* *The need for targets brings about sadness for women*

Following the qualitative analysis, we conducted a further linguistic analysis of the transcripts using a library of machine learning algorithms and natural language processing techniques to identify and extract themes, topics and emotions expressed during the interviews. The full technical details of this library are reported in its original form of an Artificial Intelligence (AI) framework,^[^ ^54^ ^]^ for the purpose of our linguistic analysis we selected a subset of these techniques, starting with data preparation that pre‐processed all interviewee responses, by excluding questions posed by the interviewer and peripheral commentary by both interviewer and interviewee to remove “noise”, “bias”, “repetition” and “offset”. The extracted data was tokenized into two subsets of data, 1) atomic syntactical units (sentences) and atomic semantic units (paragraphs).

In the next phase, these two datasets were analyzed through two pipelines of machine learning algorithms and natural language processing techniques; the first pipeline consists of keyword extraction, topic modelling, and theme parsing techniques, and the second pipeline consists of the emotion classification and sentiment analysis techniques. The second pipeline is grounded on a pre‐trained Large Language Model (LLM) that was finetuned to learn the context and nuances of how emotions are expressed within this collection of transcripts so that the emotion classification is specific to the language constructs used by interviewees when expressing themselves. The sentiment analysis algorithm classifies each sentence into positive, neutral or negative sentiment. The emotion classification technique, based on Plutchik's^[^ ^55^ ^]^ wheel of eight emotions, joy, trust, surprise, anticipation, sadness, anger, fear and disgust, classified each sentence into one of eight emotions and scored the intensity of each emotion. The keywords, topics and themes output from the first pipeline are combined to form a data‐driven ontology of the interview transcripts that can be queried, filtered and further analyzed using structured variables and output of emotion classification, emotion intensity and sentiment analysis from the second pipeline.

In the final phase, the outputs from the two pipelines are combined across corresponding granularities along with the transcript ID and corresponding structured variables. This multidimensional encapsulation keywords, topics, themes, sentiment, emotion and structured variables collected during the interview (such as age, gender, organization type) be perused using either a query language, a series of tables/spreadsheets or an interactive analytics dashboard that visualizes the intersection of themes, topics, keywords with sentiment, emotions and all other interviewee variables.

**Figure 3:**
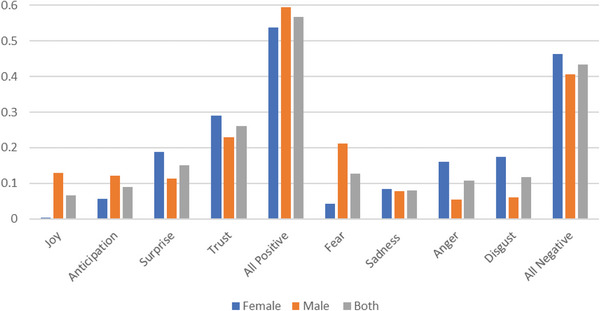
Histogram depicting the mean distribution of positive and negative emotions expressed by the interviewees.

**Table** **3** (and corresponding histogram in **Figure** **3**) shows the distribution of all the positive and negative emotion scores of the interviewee comments in the transcripts. There is not a large difference between the average emotions of men and women participants. However, the granular analysis across the spectrum of constituent emotions shows greater variation with women expressing less joy and more anger and disgust than men. These were then integrated into the coding framework as themes Figure 3.

**Table 3:** Mean distribution of positive and negative emotions expressed by the interviewees.

Gender	Count [N]	Positive emotions				
		Joy	Anticipation	Surprise	Trust	All
Female	22	0.0038	0.0561	0.1871	0.2906	0.5376
Male	19	0.1297	0.1222	0.1133	0.2290	0.5942
All	41	0.0668	0.0892	0.1502	0.2598	0.5660

Gender	Count [N]	Negative emotions				
		Fear	Sadness	Anger	Disgust	All
Female	22	0.0431	0.0835	0.1614	0.1744	0.4624
Male	19	0.2122	0.0784	0.0549	0.0602	0.4057
All	41	0.1276	0.0809	0.1082	0.1173	0.4340

## 5 Results

In the following sections we describe the results of the data as to how boards go about recruiting new board members to explore the influence of gender diversity on boards. The findings from the sentiment analysis are interwoven with the findings from the thematic analysis to provide insight into the emotions associated with the processes described.

### 5.1 Networks Limit Pool

The talent pool for potential board members largely consisted of members’ professional and social networks. Many of our male interviewees across all organizational forms described a process of member selection that involved meeting with potential new members that were in current members’ or other senior leaders’ networks. For instance:

[I was asked] would you come over and meet this guy over dinner, and it was … sort of the CEO introducing me to a potential new director and I liked him, and I thought he would add enormous value. He had great credibility, relevant sector expertise, and so on and networks. So, it was not hard at all and then the rest of the board met him. (Interview code 36D, male, chair, for‐profit)

This was a typical recruitment process where potential (male) members are introduced to current members and are informally vetted before being formally approached.

Although not very common amongst our participants, some boards employed recruitment agencies such as executive search firms to identify suitable candidates. There was a perception among some participants that recruitment firms were not very keen to assist with director searches. In this regard they adopt what we regard to be as superficial search tactics and is related to the small pipeline of potential appointees. For instance one female board member who was looking for board positions said:

I've been told by various people that appointing directors isn't overly lucrative for recruiters …. quite often, too, the board will have a couple of people in the wings … “all right we need to get somebody, a recruiter, but who knows somebody, put them into the recruitment, into the mix as part of the process”. (Interview code 32D, female board member, not‐for‐profit)

Again, it is about being the right networks, “somebody who knows somebody”.

*Use of networks to surround yourself with those similar (limit diversity)*

This type of recruitment can hinder diversity as it limits the field to those already in the social or professional business networks of existing members. People who may be qualified but are not in those networks would never be able to be approached. In addition, people tend to want to surround themselves with people like themselves, and this can obstruct recruiting for diversity, as described by two male board members:

That's your natural inclination is to be – is to have people around you that are like you. And if you're an executive and you're hiring people, you've got to always work hard to push back against your natural inclination, which is to employ someone like you. (Interview code 18D, male, board member, for‐profit and not‐for‐profit)

In terms of choosing me for that board. … I think that the two founders of that business looked at me and thought, ‘I can kind of relate to this person’. … I sort of understand him, he kind of looks kind of normal, … If I had purple hair and different socioeconomic and different gender and different political, they probably would be like nah. … In fact, I think I was actually chosen based on my lack of diversity, when I come to think of it. (Interview code 11D, male board member, not‐for‐profit)

These quotes, from directors of different types of organizations, indicate that boards are concerned with fit: how well will new members fit into the current board? Being recruited and selected is about knowing members of the board, being approached socially and accepting the invitation.

### 5.2 Few Women in Pipeline

Many participants raised the pipeline issue: that there are not enough qualified women in the pipeline to support gender diversity. One participant identified a need for “constant networking” to develop a pipeline of women:

Constant networking from the existing board members, from talking to other CEOs who are listing their company and looking at setting up boards, from being a part of the Business Council of Australia through Male Champions of Change… It's a relatively small business community in [city] (Interview code 15both, male, CEO startup).

Because of the heavy reliance on local professional networks, the perception is that without constant networking organizations struggle to attract women. The pipeline issue was frequently raised by directors, who perceived the female pool of appropriate talent was much smaller than that male pool. For example, Interviewee 1D, the male member of a not‐for‐profit board, suggested that there is a small group of women who sit on a number of boards and spoke about going back to that pool when they are seeking diversity. He said:

I know a number of women that that's what they do. And yeah, they know that there are advertisements specifically targeted to them. So, I think that kind of diversity, in the boards that I'm dealing with, is pretty easy to get. (Interview code 1D, male, board member not‐for‐profit)

While he personally had been recruited informally, there was an expectation that to find a qualified woman a board would need to advertise to attract one of the few women with experience. The appropriate women director would need to already be a director somewhere, and connected to the right networks, suggesting the importance of board interlocks to building networks, reputation and social capital for women.

This small pool of potential women candidates was also referred to a number of times by the CEOs interviewed especially those in startup companies. This is discussed by a male CEO:

Of course, the very good women are highly sought after and so they're actually far more difficult to recruit. So, you've got to make sure therefore that you're on their list well in advance of needing them and they're on your list … but if you leave it until the last minute when you've got 6 weeks and you want to make an appointment and they don't know who you are, then the high‐quality candidates won't be interested in joining. … But you may well get a qualified man because the demand and supply equations are different (Interview code 15both, male CEO, startup).

Embedded in this quote is the belief that there are few “qualified” women in the talent pool, and that it is important to work hard to attract them. Implicit is that if a firm wants to diversify they need to put forward effort, that it will not happen otherwise.

### 5.3 Specifically Seeking a Woman

Recruitment appeared to work differently for women participants, who were perceived as more difficult to attract. Indeed, there may even need to be a decision to actively recruit a woman:

… I think that, generally speaking, it is about who knows who, whose turn it is, who's in the right place at the right time, quite often, *do we need a female?* (Interview code 8IN, female, CEO).

Implicit in this quote is that the normal recruit is a man. If a woman is needed, then she will need to be specifically sought.

### 5.4 Lack of Active Recruiting to find Women Brings about Anger

The sentiment analysis found that women participants expressed higher and more intense levels of anger than men when discussing barriers to their involvement in boards, the structures that limited women's ability to be involved, the slow pace of change and the lack of active recruitment.

I've been in terrible board situations where people have been there for 35 years and things just don't change and it's awful …An accountant and I talked about this a lot too … this is a very basic example but not many women are in the finance profession so it's always easier to find a bloke who is good with the numbers because that – well, that's rubbish. It's like saying there's no female CEOs or CFOs or whatever (Interview code 23IN, female, board member and representative of not‐for‐profit directors’ body)

The quote above explicitly challenges the idea that there is an actual lack of pipeline, suggesting that there are many qualified women, if only they were to be considered. It was felt that the qualified women were somehow invisible. These quotes also identify that political will to diversify is important, and they are angry when it is not present.

### 5.5 Use of Skills Matrix Seems like a Rational Process

The skills matrix looks at the attributes required for good board governance and lists the current members’ attributes, identifying where there might be gaps. Once potential members who filled those skills gaps were identified, they were then interviewed before being selected. As described by our participants however, the application of the matrix and the interview was a more a social than rational process. The following quote shows that while the matrix might identify skills gaps, it is only one piece of information that a board considers.

I think in the first instance it's really important to get the skills matrix right so that you can be clear the skills that you are seeking, and those skills and attributes can't be, “oh well we need someone with a legal background full stop”. It has to be broader than that, hence the term “matrix”. So, you identify what you're looking for and then part of the process also needs … to look at the personal attributes of the individual as to how they will fit in with the board, and whether they are someone who is strong enough character wise to be able to hold their position on important issues but not be belligerent…

[We do this] through the interview process, the screening I suppose, … the applicants all probably look fabulous on paper but it's getting behind the documentation, checking their references and the interview. The interview is critical. And then the panel who interviews is also critical … – you've got to make sure that you don't have a panel that's really just looking for people like us. So, you've got to have diversity on your panel as well – that look at things from quite a different perspective from someone who just needs to know whether you're technically proficient (Interview code 26D, female, board member for profits).

This shows that interviews can be used to get around the more rational approach of the skills matrix, stressing the importance of diversity on the panel and the social aspects of “fit”.

One female director of an organization (who has experience as a board member of both for‐profits and not‐for‐profits) where members elect the board discussed how the board got around the need to attract women by using the skills matrix and the pipeline issue:

… they're trying really hard to vet out members who put their hands up before the election comes, so there's no competition for the people they're wanting on their board. Can you prove it? Probably not, but that's what it looks like… So, in the case, I suspect, they're looking at particular skills and backgrounds of board members, so they would probably be arguing the case that, “These are the people we need on the board.” (Interview code 37D, female board member for profits and not‐for‐profits).

So even if there was regulation to entice directors and CEOs to select women it could be circumvented by controlling the pool of candidates, limiting the pool to those deemed to have the right skills, even if they were all men. It is a seemingly rational process, but since it relies on member networks to identify candidates it can limit gender diversity.

### 5.6 Quotas not Needed‐ Women Already Appointed on Merit

Opinions about the use of quotas imposed by institutional bodies were mixed. For some, especially females the sentiment analysis demonstrated that they were sad about the lack of progress and thought that quotas would enable faster progress. For others in particular males, quotas were not needed and not helpful:

I look at the women on the Board, and they're fantastic. They don't need quotas. (Interview code 17D, male, board member for‐profits and start‐ups).

### 5.7 Targets Improve Accountability

For others, targets were an important tool:

So, one of the things that we did was as a board agree to diversity targets which we have to. We have to report them in our annual report but there's nothing that says that they have to be about board representation or there's no prescribed set of metrics. … That was a really useful mechanism for us to say, “All right, should board gender mix be an item” and I said to the board, ‘I think it should, how are we going to get there?’ There's a big push at the moment to be 30% of directors being female (Interview code 15both, male, board member of start‐ups as well as a representative of male champions of change).

For this board, formally identifying a target and being held accountable to it made them serious about recruiting women in a way they had not been previously. But worth noting this board member is a representative of one of the key institutions driving change.

The need for targets brings about sadness for women

The use of quotas was also associated with intense feelings of sadness for women in the sentiment analysis:

I used to be dead against them. I used to think that was … a race [and that] we'd evolved enough not to need targets and mandates, but now I think if we haven't evolved enough to just do the right thing then maybe we do need a rule book that says – you know, set them. Again, I've heard a few blokes go, “You know I can never get a role now because all they want is women”. I'm like, “Isn't that terrible, imagine how we felt when all they would hire is men.” “Oh yeah.” (Interview code 33D, female, board member).

Sadness was also expressed in high intensity when talking about the lack of progress being made on increasing numbers of women on boards:

The problem is most boards actually don't realize they have a problem and they don't know how to fix it … the main reason I left was culture. One of the guys was a bully, … and the chairman of course doesn't pick it up. They just don't realize how patriarchal they've been. … and so I do think the targets are important because you get them to look at it. …Gradually they sort of work it out, most of them but in organizations where they just don't have that infrastructure and they don't have that commitment coming from the top, I think it's very hard for those cultures to change. (Interview code 35D, female, board member)

The sadness uncovered by the sentiment analysis suggests a hopelessness that things can improve for women in terms of recruitment to boards. The very social process of identifying new members from existing networks makes it challenging for boards to even see they need to do their recruitment differently. For those boards that were keen to diversify, it was evident that they understood that the previous recruitment methods were not working. However, their solution was to still rely on networking, whereas an expansion of the net wider may solicit a deeper pool.

## 6 Discussion

The process described by participants for identifying potential new board members was profoundly social, shaped by the norms governing the selection process. This was the case whether there was a rational element or not, such as the use of a skills matrix or targets/quotas. The results indicate that the upper echelons of Australian organizations perceive there is a limited pipeline of qualified women for positions on boards. Even so, the process that they employ to access the pipeline and recruit new board members is one that largely limits searches to current member networks, which are male‐dominated and constrained by inequalities in corporate work (also see ref. [27]). Women participants in the research expressed anger and sadness about this situation, in comparison, men did not.

While generally interviewees spoke positively about diversity, the richness of the interview data demonstrates tensions in the practical application of such principles at board level. Some interviewees especially those representing institutions such as the Male Champions of Change and superannuation bodies spoke positively about using networks to broaden the recruitment pool, but others who were directors were more circumspect about the pool and whether gender diversity was needed at all and as such prioritized skills and experience. Directors and CEOs were often defensive in speaking about the make‐up of boards, and the recruitment of women on to boards.

Our findings are important because in Australia the discussion around the use of quotas demonstrates that it remains a long way behind other countries in appointing women onto boards. Our findings indicate that to recruit a more gender diverse board, an organization must genuinely want to diversify. Our research shows that any of the approaches to appoint women – use of networks, external recruiters, and use of skills matrices – are also barriers to recruitment. The use of networks to identify potential board members is perhaps the most limiting approach as we are most likely to be connected to those who are like us‐ through class, gender, background and skills.

In addition to our main findings, we also found that recruitment could not be considered as completely merit based. As reported above, directors and CEOs recruit new members by drawing on the networks of existing members. This was the case whether they used an external recruiter to assist with selection or not. Many boards have a low turnover, so recruitment does not happen very often. This makes the identification and selection of new members a somewhat rare and important occurrence. For a person to be identified as a potential candidate, they needed to be in the right networks, have the right background, and seem to be a viable candidate. Similar to studies in other countries^[^ ^42^, ^56^ ^]^ directors hire in their own likeness. The social perspective^[^ ^38^ ^]^ of board recruitment is apparent where potential board members reputation, networks, relationships and standing were key antecedents to being considered. Even so the limited nature of the networking does limit the pool, as others have found,^[^ ^32^ ^]^ and is evidenced through the high nature of the interlocking,^[^ ^27^ ^]^ and it is up to the directors and CEOs themselves to actively look for potential women board members from different networks. Interviewees spoke of the limited pool from which to source women but seemed to fail to develop active strategies to expand the pool and ultimately realize the benefits of diversity. Expansion of the net wider may solicit a deeper pool.

A key finding here is that the recruitment approach mattered less than the board's commitment to gender diversification. Any of the recruitment approaches could foster actual diversification of a board, the appearance of attempting to diversify (what we might call symbolic diversity efforts), or maintenance of the status quo. If a board wanted gender diversity, they could use the recruitment methods outlined to proactively bring about gender diversity. While some organizations used formal skills identification matrices, these were not always used, and could be circumvented or used as an excuse to not select women onto the board. Dobbin, Schrage and Kalev^[^ ^57^ ^]^ found that organizations who used skills tests tended to apply them to only black workers and often ignored the results for whites. Our findings show that this was similar in the selection of board members with respect to women. The matrices could be used to get the desired outcome. As Guildiken, Mallon, Fainshmidt, et al.^[^ ^25^ ^]^ argue, powerful decision makers favor current and ex‐CEOs which exclude a lot of high‐quality capable women. Moreover, skills obtained in non‐traditional employment or in non‐financial areas are not as highly valued typically.

Mc Donald and Westphal^[^ ^58^ ^]^ concluded that the “corporate elite” do not mentor potential women board members and adopt inter‐group biases to negatively impact board diversity. Groups such as the Male Champions of Change are important in bringing about such change at the organizational and board level. It was evident in this research that these institutional interviews were more active in seeking diversity. The role of directors and CEOs are also key in this process and demonstrates how they use bounded rationality in search and selection of female board members.

The research's theoretical contribution adopts the social approach to board selection and social identity theory^[^ ^26^ ^]^ as an analytical framework to explore directors and CEOs use of a social rather than rational process to board member selection that limits the recruitment of gender diverse boards. We found that no matter how board members are selected, the actual desire and resultant proactive approach to recruit for gender diversity was the most important factor in whether they were able to recruit women. It is evident that the people deciding a woman is needed are those already on the board who are predominantly men, not women. The power imbalance between the majority of male members of the board to the smaller number of female members contributed to the status quo. As outlined by Acker^[^ ^48^ ^]^ hiring through social networks is one of the ways that gender inequality is maintained. In this way they are continuing the inequalities found in organizations and legitimized through arguments that naturalized the inequality^[^ ^48^ ^]^ such as those made against the use of quotas on the basis of merit‐based recruitment. Boards and CEOs and executive search firms are basing their selection decisions on their own cognitive frames and values and recruiting in their own likeness^[^ ^27^, ^41^, ^42^ ^]^ which typically at board level include gender, career experiences, and socioeconomic roots which limits the search, and pool of candidates, and leads to the use of skills matrices that can be based on perceptions of merit and skills and which in turn can limit gender diversity. Without quotas, social networks limits board diversity and reinforces the power and status of those already on boards. Depending on the social group to which the board members are embedded, the search process can build resistance and homogeneity.^[^ ^23^ ^]^

For women the lack of progress being made and the barriers for them in accessing board roles produced high intensity of negative emotions. Sadness and anger were expressed with high intensity by women. They felt that their opportunities were being limited, and that the rate of change should have been greater. Cultural and institutional barriers were being used by males to retain the status quo as predicted by social identity theory^[^ ^26^ ^]^ and when talking about diversity, women still felt at a loss in how to bring about change. These barriers were also linked to a male view of leadership more broadly. Work at the board level continues to be organized in the image of the man who has no family responsibilities other than earning a living.^[^ ^48^ ^]^ The anger and sadness that the women felt seems to have brought a change in desired approach with some who in the past had not been in favor of quotas and mandates arguing for them to hasten the changes that are required.

## 7 Conclusion

This research contributes to the governance literature through adopting qualitative methods producing rich data from those inside the board and in key influential governance positions. This is a unique contribution as governance research has typically adopted secondary data sources due to the unwillingness of board members to be interviewed and the difficulty of accessing them. Moreover, the use of AI sentiment and emotion analysis has added a dimension to the analysis of these data sources enabling us to interrogate the interview data objectively to uncover interviewees’ emotional responses.

The key finding is boards across a variety of organizational forms are adopting a social rather than rational approach to board selection. They are using networks, recruitment agencies, skills matrices and pools of viable candidates which on the surface may appear to broaden the diversity of board members. But if they are not actively seeking gender diversity these methods can still limit diversity. Through decision‐makers prioritizing diversity in terms of skills and experiences over gender per se in their board recruitment and selection decisions these tend to limit the pool as these skills and experiences are those typically found in CEO roles and other executive roles where women are under‐represented or where there are a few women who are sought for multiple board roles. It has been found previously that women have to overcome higher barriers to obtain board positions^[^ ^35^ ^]^ and those who do not have elite backgrounds face many challenges in obtaining a boardroom position.^[^ ^59^ ^]^ It is clear that companies that are not setting targets, nor actively seeking to expand their pipelines from new networks are limiting their potential social and economic gains.

The research found that the male members who dominate the make‐up of boards do not “walk the talk” and tend to be defensive in how they operationalize the recruitment of women onto boards. They tend to go back to the established pool of candidates and that reflect their own gender, and use skills matrices to argue that those selected fill the skills gap to support decisions already made as to potential board members. They use institutional structures and established cultures to retain the status quo. This severely limits both the pipeline and the pool. They make board selection decisions based on socially constructed ideological and psychological elements that create unequal economic and power distributions.^[^ ^48^ ^]^ In this regard we found that boards need to prioritize diversity for it to be achieved. Ensuring CEOs proactively seek gender diversity, appointing interview panels that desire diversity, and ensuring the nominating committees actively seek nominations from outside established networks will all contribute to gender diverse boards.

The patriarchal culture and leadership in Australian organizations produce negative emotions by women who are board members themselves or are involved as key actors in institutional governance. The social capital of women could be utilized more broadly to build better and more representative boards and enhance decision making that is recognized as important and key to organizational success.

The lack of regulation and voluntary nature of diversity targets in Australia leaves the organization and board itself as the ultimate power in improving board diversity. As Halabi^[^ ^60^ ^]^ noted, in systems that use voluntary gender measures, the powerful influence of unconscious gender bias cannot be overcome without strong government measures. However cautionary research conducted in Norway on boards^[^ ^61^ ^]^ found that even with mandated quotas driving increased numbers of women on boards, inequality regimes continue to influence board appointments, requiring multiple‐level interventions due to historical, structural, social and economic contexts of inequality. The implications for management include the need to actively prioritize diversity in recruitment practices, going beyond traditional networks and skills matrices to include individuals from diverse professional and social backgrounds. It also highlights the patriarchal structures that dominate board recruitment, which often perpetuate gender imbalances, as well as the consideration of mandates or incentives for organizations to set gender diversity targets and expand their recruitment pipelines.

Limitations of the research are evident as the research is qualitative and therefore not generalizable. Even so the research has produced rich and detailed data, and novel and unique findings that are worthy of further exploration across other populations with different institutional contexts and through using more generalizable methods. The implications for policy are that there is a need for greater representation of women on boards and more active methods of recruitment and expansion of the networks and pools where directors are traditionally sought. Quotas may not be welcomed, but institutions such as Australian Institute of Company Directors (AICD) and ASX and the male champions of change and can drive change through increasing targets and requiring enhanced reporting. For organizations, boards and recruiters, lessons are inherent in that men who are the dominant group on boards and recruiters need to understand their efforts at what they perceive to be as rational recruitment efforts are in effect reinforcing the status quo through socially‐biased processes.

## Conflict of Interest

The authors declare no conflict of interest.

## Data Availability

The data that support the findings of this study are available from the corresponding author upon reasonable request.
